# Living/Eating Arrangement, Loneliness, and Mental Distress among Older Korean Immigrants: Gender Difference

**DOI:** 10.1093/geroni/igab046.3313

**Published:** 2021-12-17

**Authors:** Nan Sook Park, Yuri Jang, Soondool Chung, David Chiriboga, William Haley

**Affiliations:** 1 University of South Florida, Tampa, Florida, United States; 2 University of Southern California, Los Angeles, California, United States; 3 Ewha Womans University, Ewha Womans University, Seoul-t'ukpyolsi, Republic of Korea

## Abstract

Structural isolation such as living alone poses a mental health risk in diverse groups of older adults, including older immigrants. Given that those living with others might also be disengaged, the present investigation included eating alone as another source of isolation and examined the impact of the combination of living and eating alone. The proposes of the study were to examine (1) how living and/or eating alone would impact mental distress, (2) whether the impact would be mediated by feelings of loneliness, and (3) if there would be gender differences in the mediation effect. The data were drawn from the Study of Older Korean Americans (SOKA), which surveyed older Korean immigrants in five states during 2017−2018. The living/eating arrangement was classified into four-groups: living/eating with others (57%), living with others/eating alone (12.4%), living alone/eating with others (7.3%), and living/eating alone (23.1%). Using the PROCESS macro, we tested the mediation effect of loneliness and the moderation effect of gender in the relationship between the typology and mental distress controlling for background/health characteristics and social capital related variables. Two groups (living with others/eating alone and living/eating alone) had sociodemographic, health, and social capital disadvantages. Analyses demonstrated that mental distress was linked with living with others/eating alone and living/eating alone, of which relationships were mediated by loneliness only among women. Findings suggest that not only structural isolation (e.g., living alone) but also disengagement with others (e.g., eating alone) need to be considered to understand emotional well-being in older immigrant population and gender difference.

